# Idol Depletion Protects against Spontaneous Atherosclerosis in a Hamster Model of Familial Hypercholesterolemia

**DOI:** 10.1155/2022/1889632

**Published:** 2022-05-24

**Authors:** Chenxi Liang, Xiaowei Wang, Kenan Peng, Pingping Lai, Ziwei Liu, Jiaao Ma, Xin Chen, Gang Liu, Mingqi Zheng, Yuhui Wang, Hongyuan Yang, George Liu, Xunde Xian, Mingming Gao

**Affiliations:** ^1^Laboratory of Lipid Metabolism, Institute of Basic Medicine; Department of Biochemistry and Molecular Biology, College of Basic Medicine, Key Laboratory of Medical Biotechnology of Hebei Province, Cardiovascular Medical Science Center, Hebei Medical University, Shijiazhuang, Hebei 050017, China; ^2^The Key Laboratory of Neural and Vascular Biology, Ministry of Education, Shijiazhuang, Hebei 050017, China; ^3^Institute of Cardiovascular Sciences and Key Laboratory of Molecular Cardiovascular Sciences, Ministry of Education, School of Basic Medical Sciences, Peking University, Beijing, China; ^4^Department of Cardiology, First Hospital of Hebei Medical University, Shijiazhuang, Hebei 050031, China; ^5^School of Biotechnology and Biomolecular Sciences, The University of New South Wales, Sydney, NSW 2052, Australia; ^6^Beijing Key Laboratory of Cardiovascular Receptors Research, Peking University Third Hospital, Beijing 100191, China

## Abstract

Inducible degrader of low-density lipoprotein (LDL) receptor (Idol) is an E3 ubiquitin ligase coded by *Idol*, the target gene of liver X receptor (LXR), which primarily mediates the ubiquitination and lysosomal degradation of low-density lipoprotein receptor (LDLR). Previous studies from independent groups have shown that plasma cholesterol regulation by the LXR-Idol-LDLR axis is tissue- and species-specific, indicating that the precise molecular mechanism by which Idol modulates lipid metabolism has not been completely understood and needs to be further validated in other species. Hamster, a small rodent animal model expressing endogenous cholesterol ester transfer protein (CETP), possesses many metabolic characteristics that are different from mouse but similar to human. In this study, an Idol knockout (Idol^−/−^) hamster model was developed using CRISPR/Cas9 gene editing system to investigate the effect of Idol depletion on plasma lipid metabolism and atherosclerosis. Our results showed that there were no significant differences in hepatic LDLR protein and plasma cholesterol levels in Idol^−/−^ hamsters compared with wild-type (WT) controls, which was consistent with the observation that LXR agonist treatment increased the expression of *Idol* mRNA in the small intestine but not in the liver of WT hamsters. However, we found that plasma triglyceride (TG) levels were significantly reduced in Idol^−/−^ hamsters due to an enhancement of TG clearance. In addition, the morphological data demonstrated that inactivation of Idol significantly lowered plasma total cholesterol and TG levels and protected against spontaneous atherosclerotic lesions in aged LDLR knockout hamsters on a chow diet but had no effect on diet-induced atherosclerosis in hamsters lacking one copy of the *Ldlr* gene. In conclusion, our findings suggest that Idol can regulate plasma lipid metabolism and atherosclerosis independent of LDLR function.

## 1. Introduction

Hypercholesterolemia, especially elevated plasma low-density lipoprotein cholesterol (LDL-C) level, is an independent risk factor of atherosclerotic cardiovascular disease (ASCVD). Low-density lipoprotein receptor (LDLR) is expressed primarily in the liver and has been reported to be the most important protein responsible for cholesterol homeostasis by its capability of plasma LDL-C uptake through receptor-mediated endocytosis [1]. LDLR is regulated at both transcriptional and posttranscriptional levels. Low intracellular cholesterol level triggers upregulation of *Ldlr* at mRNA level by activating the sterol regulatory element binding protein-2 (SREBP-2) pathway [2], which also induces the expression of proprotein convertase subtilisin/kexin type 9 (PCSK9), a negative regulator of LDLR. After binding to the extracellular segment of LDLR, PCSK9 promotes lysosomal degradation of LDLR, leading to a decrease in LDLR protein and activity [3]. Blocking PCSK9-mediated LDLR degradation by anti-PCSK9 monoclonal antibodies has become an effective strategy for lowering LDL-C levels in patients with familial hypercholesterolemia (FH) or patients who are intolerant to statins [4].

Recently, inducible degrader of LDLR (Idol), known as myosin regulatory light chain interacting protein (Mylip), also plays a central role in posttranscriptional regulation of LDLR. Idol is an E3 ubiquitin ligase with 455 amino acids regulated by liver X receptor (LXR), which mediates ubiquitin binding enzyme 2D- (UBE2D-) dependent ubiquitination of LDLR for lysosomal degradation by binding to the cytoplasmic region of LDLR and regulates cholesterol metabolism in the manner of the LXR-Idol-LDLR axis [5–7].

Genome-wide association study (GWAS) analysis discovered that single nucleotide polymorphisms (SNPs) of Idol were related to plasma total cholesterol and LDL-C and to the responsiveness of lipid-lowering drugs [8, 9]. Sorrentino et al. found that p.Arg266X variant resulted in a complete loss of Idol function and could not promote ubiquitination and subsequent degradation of LDLR, thus leading to a prominent reduction in plasma LDL-C level [10]. Adi et al. reported that Idol p.Gly51Ser variant stabilized Idol protein through inhibiting its dimerization and self-ubiquitination to accelerate LDLR degradation and thus elevated plasma LDL-C in Chinese Uygurs [11]. Interestingly, independent studies using different experimental animal models showed contradictory results. Hong et al. have shown that LXR agonists could upregulate the expression of Idol in human and nonhuman primate hepatocytes to promote the ubiquitination degradation of LDLR, thereby increasing the level of circulating LDL-C. In addition, knocking down Idol in nonhuman primates by antisense oligonucleotide (ASO) reduced plasma LDL-C level, which was consistent with the observations of human GWAS studies. However, in mouse studies, LXR agonists induced *Idol* mRNA levels only in the macrophages and small intestine but did not affect the expression of Idol and LDLR in the liver and then LDL-C level in plasma, indicating that the LXR-Idol-LDLR axis is tissue- and species-specific [12]. Of note, although the relationship between Idol and atherosclerosis has been investigated in mice overexpressing Idol [13, 14], whether targeting Idol will yield promising results of ASCVD has not been extensively studied yet. Taken together, these data suggest that the precise molecular mechanism by which Idol regulates cholesterol metabolism and its impact on atherogenesis need to be further confirmed in other animal species.

Hamsters have been reported to be an ideal animal model for studying lipid metabolism due to the metabolic features similar to humans [15–17]. In the present study, CRISPR/Cas9 gene editing system was applied to establish a hamster model lacking Idol to investigate the effects of Idol deficiency on plasma lipid and atherosclerosis. Our results demonstrated that the LXR-Idol-LDLR axis was inactive in the liver. However, plasma triglycerides were significantly decreased in Idol knockout hamster on a chow diet, which was attributable to accelerated clearance. In addition, Idol inhibition significantly reduced plasma total cholesterol and triglyceride levels and protected against spontaneous atherosclerosis in aged LDLR knockout hamsters on a chow diet. Collectively, our findings suggest that the regulation of plasma lipid by Idol and the consequential atherosclerotic development are LDLR-independent in hamsters.

## 2. Materials and Methods

### 2.1. Animals

All the animals were housed under pathogen-free conditions in In Vivo Laboratory Animal Technology Co., LTD (Shijiazhuang, China), with a 14 h light/10 h dark cycle. For LXR agonist intervention, 12-week-old male WT and homozygous Idol knockout (Idol^−/−^) hamsters were orally administered either vehicle (0.5% carboxymethylcellulose sodium) or LXR agonist GW3965 (15 mg/kg, daily) for 7 days. To investigate diet-induced hyperlipidemia and atherogenesis, 10-12-week-old male LDLR^+/-^ and Idol^−/−^LDLR^+/-^ hamsters were fed with high-fat diet (HFD) containing 0.5% cholesterol and 10% fat for 16 weeks. All experimental procedures were in accordance with the Guide for the Care and Use of Laboratory Animals and approved by the Animal Care Committee at Peking University Health Science Center (LA2015-012).

### 2.2. Generation of Idol Knockout Hamsters

The sgRNA was designed to target the exon 2 of golden Syrian hamster *Idol* gene (NW_004801610) using Optimized CRISPR Design (http://crispr.mit.edu/). The specificity of sgRNA target sequence (GGACGGGCTGGCACCTTACAGG) was analyzed according to the NCBI BLAST applied to the golden Syrian hamster genome. The DNA template of sgRNA was amplified by PCR and then transcribed to sgRNA using MEGAscript T7 Kit (Ambion) in vitro, followed by a purification using MEGAclear Kit (Ambion). Purified sgRNA was diluted into RNase-free water at a concentration of 200 ng/*μ*L and stored at -80°C for future use. Cas9 mRNA was prepared as described previously [16]. Both sgRNA (20 ng/*μ*L) and Cas9 mRNA (50 ng/*μ*L) were coinjected into the cytoplasm of fertilized eggs with well recognized pronuclei in M2 medium and then cultured in HECM-10 medium at 37.5°C under 10% CO_2_ for 30 min. The embryos with normal morphology were then transferred into each oviduct (~15 embryos per oviduct) of surrogate hamsters that had naturally been mated with male animals one day before. For genotyping of founders, genomic DNA was extracted from the toes of individual hamster. Targeted fragments were amplified from extracted genomic DNA by PCR performed on thermal cycler (F-TTGTTTTCCCATTCTACCAC, R-TATTTCCTGAACTTCTTCTGC), and mutations were identified by Sanger sequencing. F1 generation of mutant hamsters was genotyped by PCR (F-ATATTCGCTGCCTTTGGT, R-TTGGTTTATTTCTGCCTC for *Δ*10 bp) and 4% agarose gel electrophoresis.

### 2.3. Analysis of Plasma Lipids, Lipoproteins, and Apolipoproteins

Blood samples were collected through the retroorbital vein from indicated animals fasted for 16 h under isoflurane anesthesia. The levels of plasma total cholesterol (TC), triglyceride (TG), and low-density lipoprotein cholesterol (LDL-C) were measured using enzymatic commercial kits (Bio Sino, Beijing, China). High-density lipoprotein cholesterol (HDL-C) level was measured with TC kit after precipitating apoB-containing lipoprotein by 20% polyethylene glycol (PEG).

For lipoprotein distribution analysis, pooled plasma samples from 3 to 4 hamsters per group were applied to fast protein liquid chromatography (FPLC) column (GE AKTA Purifier 100 FPLC system). The fractions were eluted at a rate of 0.5 mL per min with PBS and automatically collected. A total of 35 fractions with 0.5 mL were used to determine TC and TG in each fraction.

### 2.4. VLDL Secretion, Oral, and Intravenous Fat Load Tests

VLDL secretion assay was performed using 12-week-old hamsters. All animals were fasted for 16 h and then injected with Triton WR-1339 (MedChemExpress, Shanghai, China) at 800 mg/kg through the jugular vein. Blood samples were collected at indicated time points (baseline, 1 h, 2 h, 3 h, 4 h, and 6 h). Plasma TG levels were measured using enzymatic commercial kit (Bio Sino, Beijing, China). The VLDL-TG secretion rate was calculated from the slope of the curve between 0 and 4 h after Triton WR-1339 injection and expressed as mg/dL/hour.

For oral fat load test, 12-week-old hamsters were fasted for 16 h and then gavaged with olive oil (10 mL/kg body weight). Blood samples were collected at indicated time points (baseline, 1 h, 2 h, 4 h, 6 h, 8 h, and 11 h) to detect plasma TG concentration.

For intravenous fat load test, 12-week-old hamsters were fasted for 16 h and then injected with 20% fat emulsion at 7.5 mL/kg through the jugular vein. Blood samples were collected 1 min after fat emulsion injection as the baseline and at indicated time points after baseline (15 min, 30 min, 1 h, 2 h, 4 h, and 6 h) for plasma TG measurement.

### 2.5. Morphological Analysis

Animals were sacrificed at the endpoints of different experiments and perfused with 20 mL of 0.01 M PBS through the left ventricle. The heart and whole aorta were harvested and then fixed with 4% paraformaldehyde (PFA). Heart was embedded in OCT, snap-frozen in liquid nitrogen, and then stored at -20°C. 7 *μ*m serial sections for each sample were made from the origin of the aorta, in which all three aortic apexes were clearly observed. For the analysis of atherosclerotic lesions, lipid deposition in the whole aorta (en face) and aortic root was examined by staining with 0.5% oil red O (ORO) solution and hematoxylin. ORO-positive areas were quantified by ImageJ for atherosclerotic lesions.

### 2.6. Determination of Lipids in Stool

The lipids were extracted according to the improved method of Bligh and Dyer [18]. Briefly, 100 mg of dried stool was homogenized with 1 mL of cold 0.01 M PBS, followed by an addition of 3 mL chloroform/methanol (*v* : *v* = 2 : 1) to extract lipids. Samples were vortexed for 30 sec, then centrifuged at 1000 rpm for 20 min. The lower chloroform layer was collected after centrifugation, then dried under N_2_ flow, and finally dissolved in 3% Triton X-100 at 50°C. Enzymatic commercial kits (Bio Sino, Beijing, China) were used to measure TC and TG content.

### 2.7. Quantitative Real-Time PCR

Total RNA from the liver, small intestine, and adipose tissue was extracted with Trizol reagent (Invitrogen, USA), and the first-strand cDNA was generated with a reverse transcription (RT) kit (Promega, USA). Quantitative real-time PCR was performed to measure the expression of gene of interest (Table 1) using an Applied Biosystems with SYBR Green fluorescence (Monad, Shanghai, China). All relative gene expression levels were normalized to *Gapdh*.

### 2.8. Western Blot Analysis

Tissues were homogenized in RIPA buffer. Protein concentration was measured using BCA protein determination kit. Samples were mixed with 5× SDS sample buffer, boiled at 95°C for 10 min, and then subjected to SDS-PAGE gels. After being transferred to a nitrocellulose membrane (Applygen Technologies, Beijing, China), the proteins were identified with the indicated primary antibodies and visualized by the corresponding secondary antibodies conjugated with horseradish peroxidase and enhanced chemiluminescence detection reagent (Thermo Fisher Scientific, Shanghai, China). Protein levels were quantified with the ImageJ software. GAPDH or *β*-Actin was used as an internal control. The primary antibodies used in the study include LDLR (Abcam, England), CD36 (Proteintech, Wuhan, China), LRP1 (Abcam, England), GAPDH (Proteintech, Wuhan, China), and *β*-Actin (ABclonal, Wuhan, China).

### 2.9. Statistical Analysis

All experimental data were presented as mean ± SD. Statistical analysis was performed using the Student *t* test (comparison between two groups) or one-way ANOVA followed by the Tukey posttest using GraphPad Prism 8.0. Data that were not normally distributed were analyzed using the nonparametric Mann–Whitney *U* test. *p* value less than 0.05 was considered statistically significant.

## 3. Results

### 3.1. Generation of Idol Knockout Hamsters with Hypotriglyceridemia

**Figure 1:**
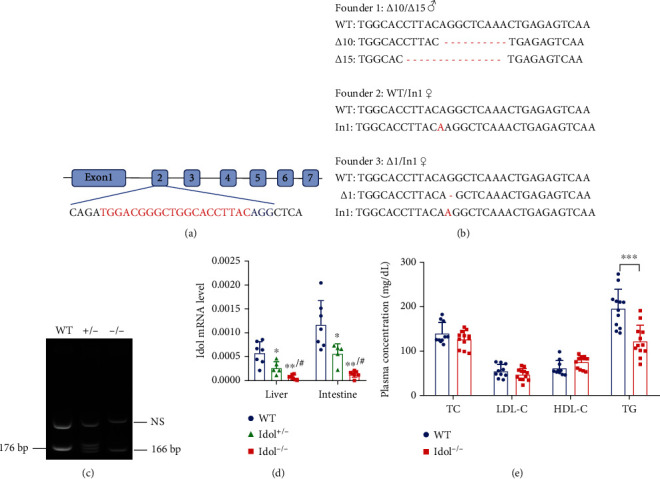
Generation of Idol knockout hamsters with hypotriglyceridemia. (a) Schematic diagram for the sgRNA targeting site at hamster Idol loci. The sgRNA target sequence is labeled in red. Letters in blue are the protospacer adjacent motif (PAM). (b) Sequencing of the targeted site in the three mutant founders. (*Δ*: deletion mutation; In: insertion mutation). (c) Genotyping of Idol knockout hamsters with 10 nt deletion. Lane 1: WT hamster (176 bp); lane 2: heterozygous Idol knockout hamster (+/-: 176 bp and 166 bp); lane 3: homozygous Idol knockout hamster (-/-: 166 bp). NS: nonspecific band. (d) Detection of *Idol* mRNA expression in the liver and small intestine. *n* = 5-7/group, ^∗^*p* < 0.05 and ^∗∗^*p* < 0.01 compared with WT; ^#^*p* < 0.05 compared with Idol^+/-^. (e) Plasma total cholesterol (TC), low-density lipoprotein cholesterol (LDL-C), high-density lipoprotein cholesterol (HDL-C), and triglyceride (TG) levels from 3-month-old male WT and Idol^−/−^ hamsters. *n* = 10-12/group, ^∗∗∗^*p* < 0.001.

In this study, sgRNA sequence was designed to target the exon 2 of hamster *Idol* gene (Figure 1(a)). We coinjected sgRNA and Cas9 mRNA into the cytoplasm of the fertilized hamster eggs and then implanted into oviducts of pregnant female hamsters. The results of DNA sequencing showed different mutations in the target site of *Idol* gene in three newborn hamster pups. As shown in Figure 1(b), there were 3 founders in total, including founder 1 with 10 nt deletion and 15 nt deletion mutations, founder 2 with 1 nt insert mutant, and founder 3 with 1 nt deletion and 1 nt insert mutation (Figure 1(b)). However, we only selected founder 1, a male hamster carrying frameshift mutant for the following studies because it was easy to genotype for breeding. Wild-type (WT) hamsters had a 176 bp band, and homozygous Idol knockout hamsters (Idol^−/−^) had a short band with 166 bp, whereas heterozygous hamsters showed double bands (Idol^+/-^) (Figure 1(c)). Next, we measured the expression of hepatic and intestinal *Idol* mRNA and found that it was undetectable in Idol^−/−^ hamsters by quantitative real-time PCR (Figure 1(d)), suggesting that the *Idol* gene was successfully deleted from hamster by CRISPR/Cas9 gene editing. To study the effect of Idol deficiency on plasma lipid metabolism in hamsters, we determined the plasma lipid levels of WT and Idol^−/−^ hamsters fed with a standard rodent chow diet and found that plasma TC, LDL-C, and HDL-C levels of Idol^−/−^ hamsters were not different from WT hamsters, while circulating TG was significantly decreased by 37% and 34% in male and female Idol^−/−^ hamsters, respectively (Figure 1(e) and Suppl Figure 1). These results indicated that Idol deficiency did not affect plasma cholesterol levels but significantly reduced the plasma triglyceride level in hamsters without any dietary intervention.

### 3.2. Increased LDLR Protein Level in the Intestine of Idol^−/−^ Hamsters

**Figure 2:**
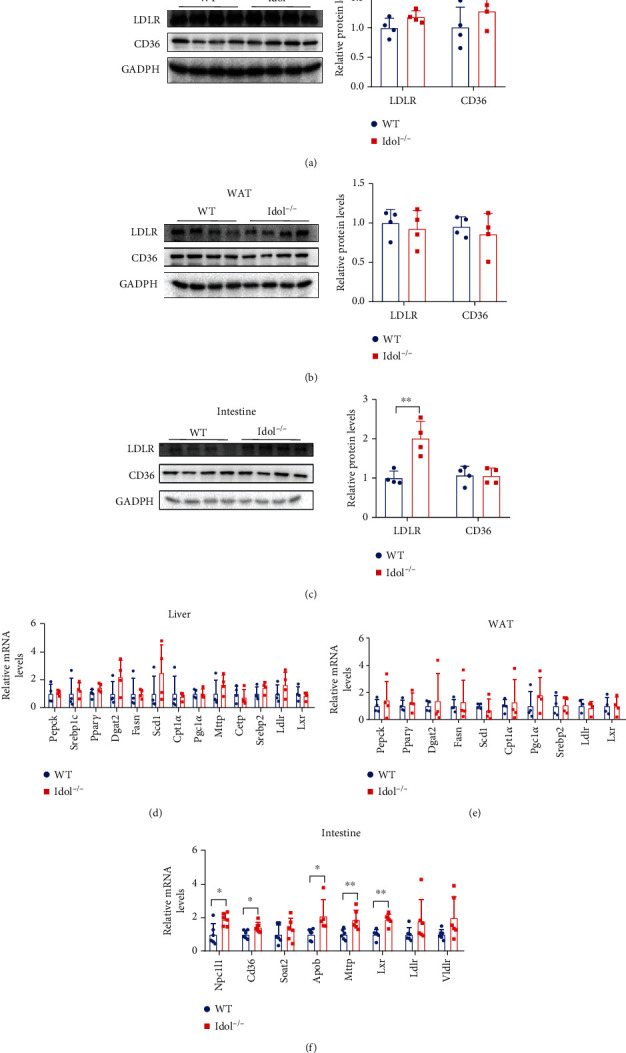
Tissue-specific modulation of LDLR expression in WT and Idol^−/−^ hamsters. (a–c) Western blotting and quantitative analysis of LDLR and CD36 protein expression in the liver (a), white adipose tissue (b), and small intestine (c). *n* = 4/group, ^∗∗^*p* < 0.01. (d–f) Relative mRNA expression levels of genes regulating lipid metabolism in the liver (d), white adipose tissue (e), and small intestine (f). *n* = 4/group in (d) and (e); *n* = 6-7/group in (f).

To investigate whether targeting Idol influenced LDLR regulation in different tissues of hamsters, we detected LDLR protein in the liver, white adipose tissue (WAT), and small intestine. Unexpectedly, we did not find notable changes in LDLR protein levels in the liver and WAT in Idol^−/−^ hamsters when compared with WT hamsters, suggesting that Idol deletion did not significantly regulate LDLR protein in the liver of hamsters (Figures 2(a) and 2(b)), which was consistent with the unaltered plasma cholesterol levels. However, similar to the results reported from mice, LDLR protein levels in the small intestine of Idol^−/−^ hamsters were significantly increased (Figure 2(c)). Although Idol^−/−^ hamsters did not show any changes in hepatic LDLR protein and plasma cholesterol levels, surprisingly, we found that the plasma TG levels of Idol^−/−^ hamsters were markedly reduced, indicating that Idol might regulate triglyceride metabolism through other unknown pathways independent of LDLR function. Moreover, previous studies reported that PCSK9 could regulate the expression of CD36 to affect long-chain fatty acid uptake and triglyceride metabolism in the liver and adipose tissue of mice [19]. In our hamster model, we found that CD36 expression levels in the liver, WAT, and small intestine of WT and Idol^−/−^ hamsters were identical (Figures 2(a)–2(c)). In addition, we also detected mRNA expressions of genes involved in the regulation of lipid metabolism in the liver, WAT, and small intestine, and no significant changes were observed in liver and WAT (Figures 2(d) and 2(e)). However, the expression levels of genes modulating lipid absorption and chylomicron assembly, such as *Npc1l1*, *Cd36*, *Apob*, and *Mttp*, were upregulated in the small intestine (Figure 2(f)).

### 3.3. Effects of LXR Agonist on Idol Expression and Lipid Metabolism in Hamsters

**Figure 3:**
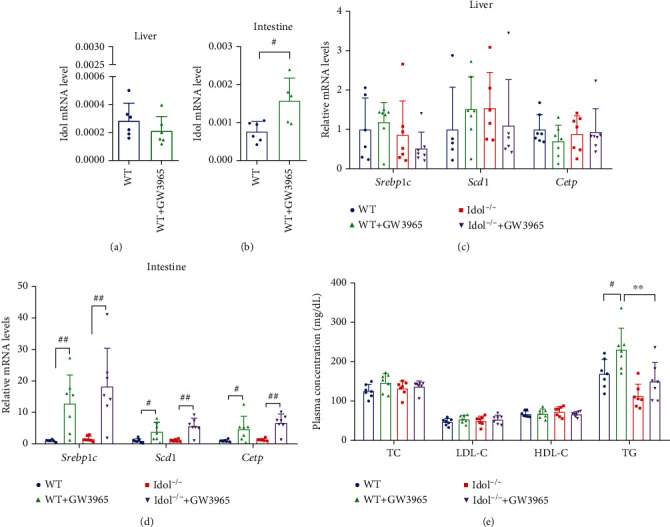
Effects of LXR agonist (GW3965) on Idol expression and lipid metabolism in hamsters. (a, b) The effect of GW3965 on the expression of Idol in the liver (a) and small intestine (b). *n* = 5-7, ^#^*p* < 0.05. (c, d) The effect of GW3965 on the mRNA expression of *Srebp1c*, *Scd1*, and *Cetp* in the liver (c) and small intestine (d). *n* = 5-7, ^#^*p* < 0.05; ^##^*p* < 0.01. (e) The effect of 7-day oral administration of GW3965 (15 mg/kg) on plasma TC, LDL-C, HDL-C, and TG levels. *n* = 7/group, ^#^*p* < 0.05; ^∗∗^*p* < 0.01.

To explore the regulatory effect of LXR on Idol in hamsters, we treated WT and Idol^−/−^ hamsters with LXR agonist GW3965 for 7 days and found that LXR agonist could significantly upregulate the mRNA level of *Idol* in the small intestine of WT hamsters but had no effect on hepatic *Idol* (Figures 3(a) and 3(b)). In addition, we found that GW3965 significantly upregulated the mRNA expression of gene regulating lipid metabolism, such as *Srebp1c*, stearoyl-CoA desaturase 1 (*Scd1*), and cholesterol ester transporter protein (*Cetp*) in the small intestine. However, the expression of these genes in the liver was not significantly affected (Figures 3(c) and 3(d)). These results indicated that LXR had little effects on *Idol* and other lipid metabolism-related genes in the liver of hamsters. Meanwhile, we also determined plasma lipid levels of GW3965-treated WT and Idol^−/−^ hamsters and our results showed an increase in plasma TG levels in both WT and Idol^−/−^ hamsters, but no significant changes in plasma cholesterol levels. Interestingly, plasma TG concentration of GW3965-treated Idol^−/−^ hamsters was still lower than that of WT hamsters with the same treatment (Figure 3(e)), suggesting that LXR agonists modulate triglyceride metabolism by Idol in hamsters, which had not been discovered yet in other species.

### 3.4. Accelerated TG Clearance in Idol^−/−^ Hamsters

**Figure 4:**
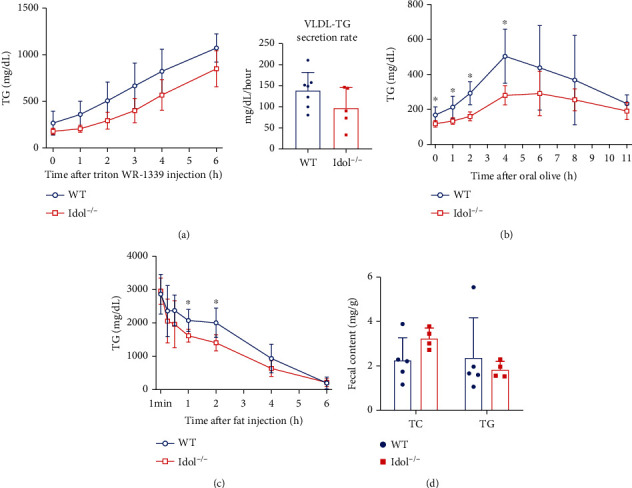
Triglyceride metabolism in Idol^−/−^ hamsters. (a) VLDL secretion analysis was performed in animals after intravenous injection of Triton WR-1339 (800 mg/kg). *n* = 5-7/group. (b) Oral fat load test was conducted in animals after olive oil gavage (10 mL/kg). *n* = 4-6/group, ^∗^*p* < 0.05. (c) Plasma triglyceride clearance was analyzed in animals after intravenous injection of 20% intralipid (7.5 mL/kg). *n* = 7/group, ^∗^*p* < 0.05. (d) Fecal cholesterol and triglyceride contents. *n* = 4-5/group.

To better understand the mechanism by which Idol deficiency reduced plasma TG in hamsters, we examined hepatic VLDL secretion, intestinal TG absorption, and blood TG clearance. First, Triton WR-1339 was injected intravenously to inhibit lipoprotein lipase- (LPL-) mediated TG hydrolysis process in circulation, thus representing TG production from hepatic VLDL secretion. Although plasma TG levels at the indicated time points in Idol^−/−^ group were lower than those in the control group, the secretion rate (slope) was identical, indicating that hepatic VLDL secretion rate in Idol^−/−^ hamsters was not different from WT hamsters (Figure 4(a)). Next, we performed an oral fat load test on WT and Idol^−/−^ hamsters by determining TG concentration at different time points after olive oil gavage, which reflected the combined effect of intestinal TG absorption and plasma TG clearance from circulation. In the first 2 hours after olive oil gavage, TG levels in Idol^−/−^ hamsters were significantly lower than those in the WT control group, suggesting reduced absorption of TG in the small intestine or accelerated plasma clearance (Figure 4(b)). Afterward, the intravenous fat load test was conducted to determine the capacity of plasma TG clearance by detecting TG concentration at different time points after intravenous lipid emulsion injection. We found that TG levels of Idol^−/−^ hamsters were also lower than those of the WT genotype, of which the difference between 1 h and 2 h was the most significant, indicating an increase in the capacity of plasma TG clearance in Idol^−/−^ hamsters (Figure 4(c)). Moreover, we found that there were no significant differences in the contents of TG and TC in feces between two groups, implying that the lipid absorption capacity of the small intestine was similar between WT and Idol^−/−^ hamsters (Figure 4(d)). Collectively, these results suggested that reduced plasma TG levels in Idol^−/−^ hamsters were probably attributed to an acceleration of TG clearance from circulation.

### 3.5. Diet-Induced Atherosclerosis in Heterozygous LDLR-Deficient (LDLR^+/-^) Hamsters with Idol Deficiency

**Figure 5:**
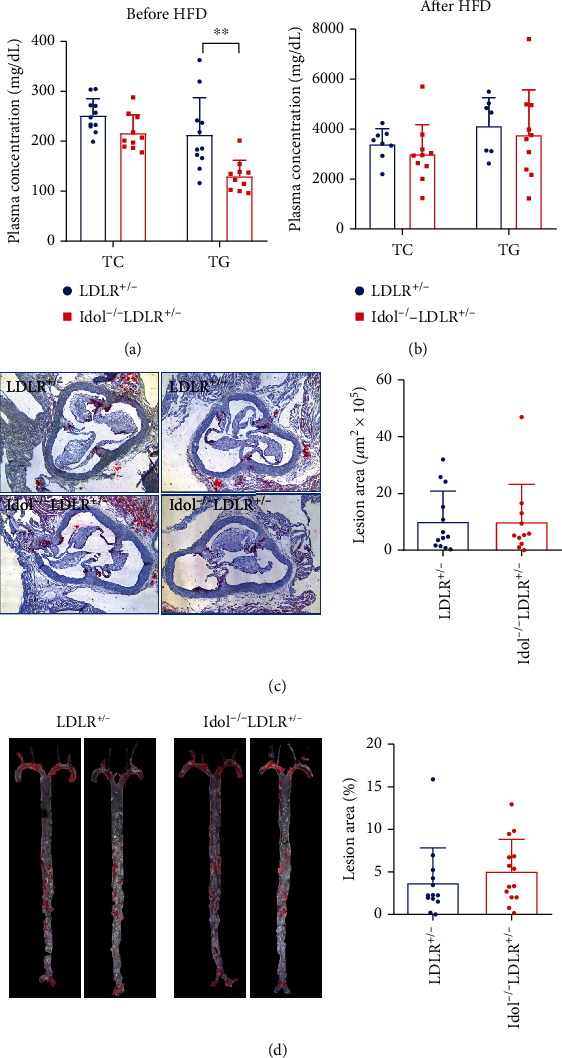
HFD-induced hyperlipidemia and atherosclerosis in Idol^−/−^LDLR^+/-^ hamsters. (a, b) Plasma cholesterol and triglyceride levels of LDLR^+/-^ and Idol^−/−^LDLR^+/-^ hamsters before (a) and after (b) high-fat diet (HFD) for 16 weeks. *n* = 8-10/group. (c) Morphological and quantitative analysis of ORO-positive atherosclerotic lesion areas in the aortic roots of the indicated animals in (b). *n* = 11/group. (d) Morphological and quantitative analysis of ORO-positive atherosclerotic lesion areas in the whole aortas of the indicated animals in (b). *n* = 13/group.

Since loss of Idol differentially affected cholesterol and TG in chow-fed hamsters and LDLR^+/-^hamsters were susceptible to diet-induced hyperlipidemia and atherosclerosis, we next developed Idol^−/−^LDLR^+/-^ hamsters fed a high-fat diet (HFD) containing 10% lard and 0.5% cholesterol for 16 weeks to study the impact of Idol on hyperlipidemia and atherogenesis under the condition of dietary intervention. Plasma TG levels were significantly reduced in Idol^−/−^LDLR^+/-^ hamsters when compared to LDLR^+/-^ hamsters before HFD feeding (Figure 5(a)); however, plasma TC and TG levels were significantly increased in both two groups after HFD feeding, but there were no significant differences in lipid levels between the two genotypes (Figure 5(b)). Consistently, HFD-fed Idol^−/−^LDLR^+/-^ and LDLR^+/-^ hamsters showed identical atherosclerotic lesion areas in the aortic roots and the whole aortas (Figures 5(c) and 5(d)), suggesting that inactivation of Idol did not affect diet-induced hyperlipidemia and atherosclerosis in the presence of one copy of *Ldlr* in hamsters.

### 3.6. Improved Hyperlipidemia and Spontaneous Atherosclerosis in Chow-Fed Idol^−/−^ Hamsters in the Absence of LDLR

**Figure 6:**
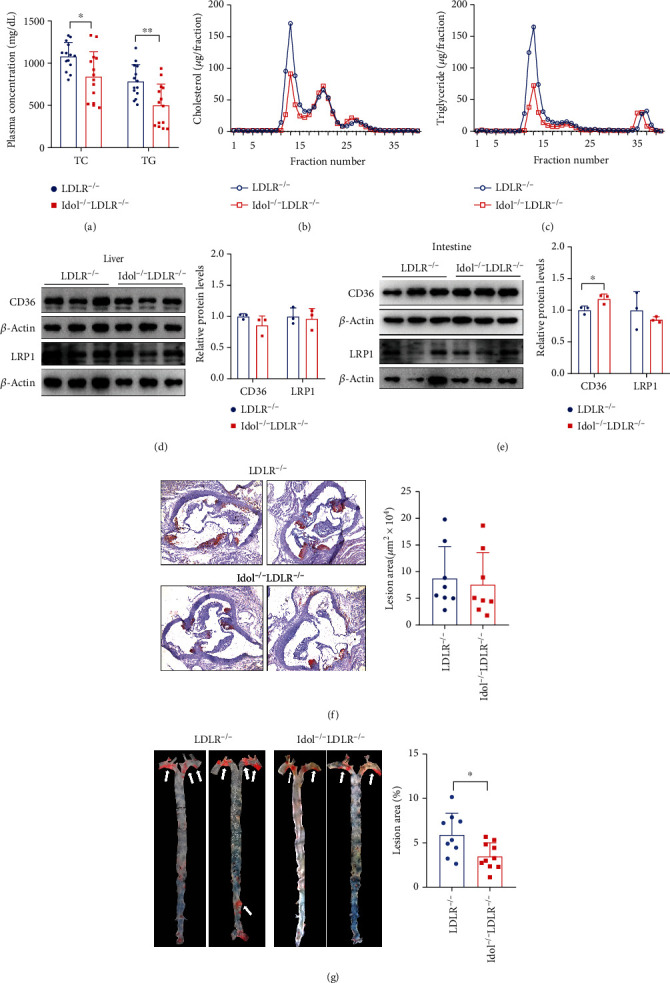
Lipid metabolism and spontaneous atherosclerosis in Idol^−/−^LDLR^−/−^hamsters on chow diet. (a) Plasma cholesterol and triglyceride levels of 6-month-old LDLR^−/−^ and Idol^−/−^LDLR^−/−^ hamsters on chow diet. *n* = 14/group, ^∗^*p* < 0.05, ^∗∗^*p* < 0.01. (b, c) Analysis of plasma lipoprotein distribution. Cholesterol (b) and triglyceride (c) contents in different fractions of pooled plasma from 6-month-old LDLR^−/−^ and Idol^−/−^LDLR^−/−^ hamsters were determined. *n* = 6/group. (d, e) CD36 and LRP1 protein expression of the liver (d) and small intestine (e). *n* = 3/group, ^∗^*p* < 0.05. (f) Morphological and quantitative analysis of ORO-positive atherosclerotic lesion areas in the aortic roots from 18-month-old hamsters *n* = 8/group. (g) Morphological and quantitative analysis of ORO-positive atherosclerotic lesion areas in the whole aortas of the indicated animals in (f). *n* = 9-10/group, ^∗∗^*p* < 0.01.

Considering that Idol may function through an LDLR-independent pathway, we crossed Idol^−/−^ hamsters with homozygous LDLR-deficient hamsters to obtain homozygous double mutant (Idol^−/−^LDLR^−/−^) hamster model. Surprisingly, Idol^−/−^LDLR^−/−^ hamsters displayed an overt reduction in both plasma TC and TG levels when compared to LDLR^−/−^ hamsters (Figure 6(a)). Fast protein liquid chromatography (FPLC) results showed that the contents of cholesterol and TG in the VLDL fractions were significantly decreased (Figures 6(b) and 6(c)). Next, we examined the levels of CD36 and LRP1, two key receptors responsible for lipoprotein metabolism, in the liver and small intestine, and our data showed that the protein level of CD36, but not LRP1, in the small intestine of Idol^−/−^LDLR^−/−^ hamsters was significantly increased relative to LDLR^−/−^ controls (Figures 6(d) and 6(e)), indicating that Idol could regulate plasma lipid through the CD36 signaling pathway in the absence of LDLR. As our previous study discovered that spontaneous atherosclerosis was developed in aged LDLR^−/−^hamsters fed with a normal rodent diet [15], we investigated atherosclerotic lesions on the aortic roots and whole aortas of 12~18-month-old Idol^−/−^LDLR^−/−^ hamsters without any nutrient intervention. The morphological results from oil red O (ORO) staining showed that although the atherosclerotic lesion areas of aortic roots were similar in the two groups (Figure 6(f)), the lesions in whole aortas of Idol^−/−^LDLR^−/−^ hamsters were significantly decreased compared with those of the LDLR^−/−^ control group (Figure 6(g)). Taken together, these results demonstrated that Idol inhibition could mitigate hyperlipidemia and protect against spontaneous atherosclerosis in LDLR^−/−^ hamsters.

## 4. Discussion

The E3 ubiquitin ligase Idol mediates ubiquitination and lysosomal degradation of LDLR, and its SNPs are closely associated with plasma LDL-C levels in humans [5, 8]. It has been shown that the regulation of the LXR-Idol-LDLR axis is active in the liver of primates; however, in mice, neither LXR agonist nor knocking out Idol affects hepatic LDLR protein expression and plasma LDL-C levels, indicating that the LDLR regulation by LXR/Idol pathway is species- and tissue-specific [12]. In this study, we proposed that Syrian golden hamster, another small rodent animal replicating the characteristics of human lipid metabolism, would be an alternative animal model to study the physiological functions of Idol *in vivo*. Surprisingly, our results showed that the hepatic LXR-Idol-LDLR axis was inactive in hamsters, which was in agreement with the observations in mice. However, plasma levels of TG, but not cholesterol, were significantly reduced in Idol^−/−^ hamster probably due to an accelerated clearance. In addition, Idol deficiency significantly ameliorated combined hyperlipidemia with elevated total cholesterol and TG levels and protected against spontaneous atherosclerosis in aged LDLR knockout hamster. Our results indicate that Idol could regulate plasma lipid and atherosclerosis independent of LDLR function in hamsters.

Prior work has clearly demonstrated that the Idol/LXR pathway is the major determinant of LDLR function in mouse intestine, but not mouse liver, which is distinct from humans and NHPs, suggesting that a more humanized mouse model be considered. Since both humans and Syrian golden hamsters possess the high similarities in lipid metabolism, such as endogenous cholesteryl ester transfer protein (CETP) and intestine-only ApoB editing, it was made an ideal animal model used for lipid study [20–22]. In our study, hamsters lacking Idol showed increased LDLR protein levels only in the intestine, but not in the liver and WAT without affecting cholesterol levels in circulation, which is consistent with the findings observed in mice, indicating that CETP may have no impact on LXR/Idol-mediated LDLR regulation and then cholesterol metabolism in small rodent animal models. Interestingly, Ibrahim et al. reported that overexpression of human Idol by adenoassociated virus (AAV) in a “humanized” mouse model, LDLR^+/-^/Apobec1^−/−^/hApoB-Tg mouse showing ApoB100-only lipoproteins, significantly reduced hepatic LDLR protein and increased plasma LDL-C, supporting the concept that CETP is not involved in the LXR-Idol-LDLR pathway [14]. Moreover, it should be noted that inactivation of Idol in chow-fed homozygous FH hamsters with hypercholesterolemia could markedly lower plasma cholesterol levels, demonstrating that targeting Idol may attenuate elevated cholesterol levels in circulation but has no effect on normal plasma cholesterol levels in hamsters. However, it will be tempting to investigate the mechanism by which Idol regulates cholesterol metabolism in the setting of FH in the future.

Although the plasma cholesterol levels were identical in Idol^−/−^ and WT hamsters, the former unexpectedly showed reduced TG levels and the TG lowering effect was still prominent in Idol^−/−^LDLR^−/−^ hamsters. Our data demonstrated that intestinal-derived TG-rich lipoproteins were reduced at least partially though the CD36 signaling pathway contributing to this beneficial effect because CD36, an important regulator of fatty acid uptake and oxidation, was significantly increased in the intestine. Consistent with our findings, Drover and colleagues have shown that CD36 deficiency impairs intestinal lipid processing, leading to secretion defect and then hypertriglyceridemia [23], and another group led by Ibrahimi discovered that overexpressing CD36 could reduce plasma TG [24]. Thus, how Idol modulates CD36 function and TG metabolism in hamsters in the presence or absence of LDLR will clearly need to be validated.

Previous studies have shown that transgenic mice overexpressing human Idol developed visible atherosclerotic lesions [13, 25]; however, whether genetical or pharmaceutical inhibition of Idol could yield a promising outcome of ASCVD has not been fully addressed yet. As it has been reported that patients with heterozygous FH show an increase by 20-fold in the incidence of ASCVD without any medication intervention [26] and heterozygous LDLR-deficient hamsters are predisposed to diet-induced atherosclerosis [15], we crossed Idol^−/−^ into LDLR-deficient background to generate Idol^−/−^LDLR^+/-^ hamsters, which were fed with HFD for 16 weeks. After HFD challenge, there were no significant differences in atherosclerotic lesions between two genotypes. We speculated that severe combined hyperlipidemia caused by HFD feeding could override the beneficial effects of Idol inhibition on lipid metabolism to exacerbate diet-induced atherosclerotic development. To exclude the possibility that severe hyperlipidemia is the major culprit of diet-induced atherogenesis, we applied homozygous FH hamsters, an animal model with hypercholesterolemia (~1000 mg/dL) and atherosclerosis under chow diet condition, to our present study. As expected, depletion of Idol significantly reduced plasma TC and TG levels to generate an antiatherogenic lipoprotein profile and then decreased atherosclerotic lesions in chow fed-homozygous FH hamsters, demonstrating that Idol could be a potential therapeutic target for ASCVD.

In summary, we developed a hamster model with Idol deficiency for the first time to investigate the role of Idol in lipid metabolism and atherosclerosis. Our data demonstrate that Idol can regulate TG metabolism through the CD36 signaling pathway in hamsters and inactivation of Idol attenuates hyperlipidemia and protects against spontaneous atherosclerosis in setting of FH, suggesting that Idol will be a potential target for the treatment of lipid disorder and ASCVD.

## Figures and Tables

**Table 1:** A list of primers for qPCR.

Gene	Forward	Reverse
*Apob*	AGATGCCAACCTGGATTTCTTA	CCATATGGAGAAATCCTTCAGC
*Cd36*	GTATTCTCATGCCAGTTGGAGAC	TTTAACCCAGTTTTTGAAAGCAA
*Cetp*	AGAATTCCTCTTCCCACATAAGG	GGAAATCGCTAAGGCTTAAGAAG
*Cpt1α*	TCTTCAAAAACAGCAAGATAGGC	GGGTTGGTTTCTCCTTTACAATG
*Dgat2*	TACAAGCAGGTAATCTTTGAGG	GGGCAAAACCAATATACTTCTG
*Fasn*	GCAGTCTTGAGTAGCTTTGTGCT	GGGAGCTGTCCAGATTAATACCT
*Gapdh*	ACTGATGCCCCCATGTTTGT	TGCAGGCAAAGTTATCCCACT
*Idol*	CTGGCACCTTACAGGCTCAA	TCTCCGAATTTGGTCTGGGC
*Ldlr*	GCATCACACTAGATATTCCCAGT	GAGTTTGGAATCAACCCAATAGA
*Lxr*	GTCCACAAAAGCGGAAAAAG	CTCGCAGCTCAGAACAATGTA
*Mttp*	AGAGGAAAACCTGGACTCCTATG	AGCATTTTGGACATCAGATCACT
*Npc1l1*	AGAAGATCCAATATGCCACTGAA	TATTGGGGCCAAAGATATAAAGG
*Pepck*	GGCTGGCTATCAGGCACAG	TGCAGTTGAGGAGGCATGTT
*Pparα*	GTGGCTGCTATAATTTGCTGTG	AGCTTCGGGAAGAGAAAGGTAT
*Pparg*	CAATCAAAGTAGAACCTGCATCC	TAGTGGAAGCCTGATGCTTTATC
*Scd1*	ATTACTGGAGTGAAGCTTTCGTG	GATTCAATGTTCTTGTCGTAGGG
*Srebp1c*	GCGGACGCAGTCTGGG	ATGAGCTGGAGCATGTCTTCAAA
*Srebp2*	AGTTGGCAAACCAAAAAAACAAG	GATTAAAGTCTTCAATCTTCAAGTCCAC
*Soat2*	GGCGAGTGTTCAGGTCATCA	GCCGCGATCATCAGAGGTAA
*Vldlr*	CGAGAGTGTCAAAGGATCAATGTG	GTGGGGTGCTACTGGTTCAG

## Data Availability

The data used to support the findings of this study are included within the article.

## Supplementary Materials

Supplementary Materials Suppl Figure 1: plasma total cholesterol (TC), low-density lipoprotein cholesterol (LDL-C), high-density lipoprotein cholesterol (HDL-C), and triglyceride (TG) levels from 3-month-old female WT and Idol^−/−^ hamsters on chow diet.
