# Supplementary material for: Idol Depletion Protects against Spontaneous Atherosclerosis in a Hamster Model of Familial Hypercholesterolemia
Source: Oxid Med Cell Longev. 2022 May 24;2022:1889632. doi: 10.1155/2022/1889632 (PMC9155911; doi:10.1155/2022/1889632)
Supplement: Supplementary Materials — Suppl Figure 1: plasma total cholesterol (TC), low-density lipoprotein cholesterol (LDL-C), high-density lipoprotein cholesterol (HDL-C), and triglyceride (TG) levels from 3-month-old female WT and Idol−/− hamsters on chow diet. [file 1889632.f1.docx]

**Supplemental figure and figure legend**

**
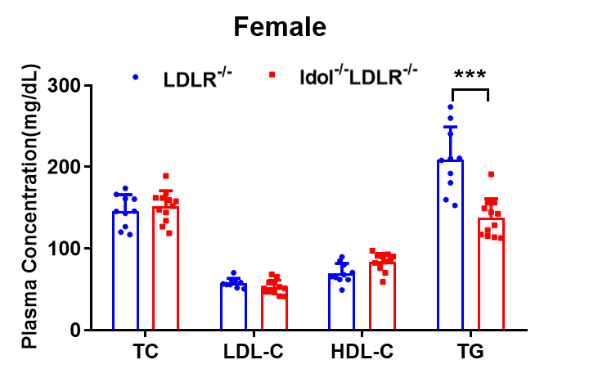
**

Suppl Figure 1: Plasma total cholesterol (TC), low density lipoprotein cholesterol (LDL-C), high density lipoprotein cholesterol (HDL-C) and triglyceride (TG) levels from 3-month old female WT and Idol^-/-^ hamsters on chow diet. n=10~12/group, *** *p* <0.001.
